# C-752-04. Perioperative Hypothermia in Burn Surgery: Current Management Strategies and the Role of Esophageal Warming

**DOI:** 10.1093/jbcr/irag033.017

**Published:** 2026-04-06

**Authors:** Shelby Harris, Susan L Smith, Michael L Cheatham

**Affiliations:** Warden Burn Center at Orlando Health, Orlando Regional Medical Center, Orlando, FL; Warden Burn Center at Orlando Health, Orlando Regional Medical Center, Orlando, FL; Warden Burn Center at Orlando Health, Orlando Regional Medical Center, Orlando, FL

## Abstract

**Introduction:**

Perioperative hypothermia (< 36°C) in burn patients increases risks of blood loss, infection, graft loss, and prolonged length of stay. Burn patients are uniquely predisposed due to impaired thermoregulation, large wound exposure, and prolonged operative times. Despite these risks, practice variation persists. We sought to identify perioperative factors associated with hypothermia and evaluate the impact of passive and active warming strategies, including esophageal heat exchange devices (EHEDs).

**Methods:**

We retrospectively reviewed adult burn patients undergoing inpatient operative intervention from 2020–2024. Procedures <1 hour, outpatient cases, and non-burn operations were excluded. Demographics, comorbidities, injury characteristics, intraoperative warming devices, medications, and fluid/blood product use were analyzed. Outcomes included incidence of perioperative hypothermia and associations with patient and management variables. Intravascular temperature management systems were not utilized.

**Results:**

Overall, 41% of patients developed perioperative hypothermia. Hypertension was significantly protective (*p*<.05). Hypothermia correlated with larger TBSA burns, longer operative times, vasopressor use, and increased crystalloid and blood product administration. EHED use reduced hypothermia rates to 18% vs. 46% without. Patients managed with EHEDs maintained higher mean intraoperative temperatures (36.4°C vs. 35.6°C, *p*<.01). Intraoperative nadir <35°C occurred in 4% without EHEDs vs. < 1% with. On analysis, EHED patients had significantly longer operative times (235 vs. 126 min) and longer ICU and hospital stays (20 vs. 3.6 days; 82 vs. 17 days) compared with non-users. Maintaining comparable or improved thermal control in this highest-risk cohort suggests EHEDs provided protection in the sickest, longest cases.

**Conclusions:**

Perioperative hypothermia remains common in burn surgery, influenced by TBSA, resuscitation requirements, and intraoperative management. EHEDs provided superior maintenance of normothermia and reduced severe hypothermia, supporting their role in multimodal warming. Our review highlighted significant inconsistencies in practice, prompting development of an institutional Clinical Practice Guideline for the Prevention of Perioperative Hypothermia in Burn Patients. Standardized multimodal warming, environmental controls, and monitoring aim to reduce hypothermia-related complications and improve outcomes.

**Applicability of Research to Practice:**

Our findings show that EHEDs help maintain normothermia even in the longest, highest-risk burn cases. Incorporating these results into standardized multimodal warming protocols, as adopted in our institutional guideline, offers a practical strategy to reduce hypothermia and improve outcomes.

**Funding for the study:**

N/A.

